# Elevated serum Meteorin-like levels in patients with hyperthyroidism

**DOI:** 10.1186/s12902-022-01229-7

**Published:** 2022-12-07

**Authors:** Xiaohui Wen, Xiaoyu Ding, Xiaona Chang, Jiaxuan Wang, Qiu Wang, Jia Liu, Guang Wang

**Affiliations:** 1 Department of Otolaryngology Head & Neck Surgery, Beijing Chao-Yang Hospital, Capital Medical University, Beijing, 100020 China; 2 Department of Endocrinology, Beijing Chao-Yang Hospital, Capital Medical University, NO. 8, Gongti South Road, Chaoyang District, Beijing, 100020 China

**Keywords:** Metrnl, Hyperthyroidism, Thyroid hormones, Metabolism

## Abstract

**Background:**

Meteorin-like (Metrnl) is a newly discovered adipomyokine that regulates systemic energy homeostasis. Both thyroid hormones and Metrnl increase energy expenditure and induce browning of adipose tissue. Thus, the aim of this study was to investigate serum Metrnl levels in hyperthyroid patients and the association of serum Metrnl levels with hyperthyroidism.

**Methods:**

The study included 88 patients with newly diagnosed untreated overt hyperthyroidism and 100 age- and sex- matched healthy controls. Serum Metrnl levels were determined using the enzyme-linked immunosorbent assay (ELISA) method.

**Results:**

Serum Metrnl levels were significantly elevated in patients with hyperthyroidism compared with controls. Linear regression analyses indicated that serum Metrnl levels were independently associated with FT3 (*β* = 0.324, *P* = 0.001), FT4 (*β* = 0.293, *P* = 0.001), and TSH (*β* = -0.234, *P* = 0.006) after full adjustment. Additionally, further logistic regression analyses revealed that the highest Metrnl tertile was significantly associated with hyperthyroidism compared with the lowest tertile (*P* for trend < 0.001). The relationship remained significant even after adjusting for potential confounders. Meanwhile, each one-unit increase in circulating Metrnl was independently associated with hyperthyroidism (OR 1.021, 95%CI 1.007–1.036, *P* < 0.01).

**Conclusion:**

Serum Metrnl levels were elevated in patients with hyperthyroidism and were independently associated with hyperthyroidism.

## Introduction

Thyroid hormone (TH) is a key regulator of energy homeostasis responsible for normal growth, development and metabolism [1, 2]. It is well established that TH maintains basal metabolic rate, promotes adaptive thermogenesis, and regulates body weight by fine-tuning food intake and energy expenditure [3, 4]. Hyperthyroidism, excess TH, presents a hypermetabolic condition characterized by increased energy expenditure, weight loss, heat intolerance, reduced cholesterol levels, and accelerated lipolysis [5]. TH modulates metabolism primarily via binding to thyroid hormone receptor (TR) α or β, acting on the brain, white adipose tissue (WAT), brown adipose tissue (BAT), skeletal muscle, and liver [5]. Of note, animal and human studies have shown that excess TH stimulates BAT activity and induces browning of adipose tissue by increasing uncoupling protein-1 (UCP-1) gene expression [6–9]. Besides, recent studies have reported that thyroid dysfunction can affect circulating levels of several cytokines, such as irisin [10], fibroblast growth factor 21 (FGF21) [11], and neuregulin 4 (Nrg4) [12], suggesting that TH interacts with cytokines secreted from adipose tissue, skeletal muscle or liver to modulate whole-body metabolism. However, the mechanism of metabolic regulation in hyperthyroidism is complicated and not fully elucidated.

Meteorin-like (Metrnl), a recently identified adipomyokine, is synthesized and secreted mainly by adipose tissue and skeletal muscle upon stimulation by cold exposure and exercise, respectively [13, 14]. Rao et al. found that increases in circulating Metrnl promoted energy expenditure and stimulated adipose tissue browning by increasing the expression of UCP-1, type 2 deiodinase (DIO2), peroxisome proliferator‐activated receptor gamma (PPARγ) coactivator 1‐alpha (PGC‐1α), and other thermogenic genes [13]. Furthermore, Metrnl reduces high fat diet (HFD)-induced body weight gain and improves insulin resistance via AMP-activated protein kinase and PPARδ-dependent pathways in skeletal muscle [15]. Both TH and Metrnl are involved in regulating energy expenditure and browning of adipose tissue. Due to numerous similarities in action, it seems imperative to explore these substances’ potential mutual influence on the body. We, therefore, aimed to investigate serum Metrnl levels in hyperthyroid patients and the association of serum Metrnl levels with hyperthyroidism.

## Materials and methods

### Study population

This cross-sectional study recruited 88 patients with newly diagnosed untreated overt hyperthyroidism who were examined at the outpatient clinic of Beijing Chao-Yang Hospital from October 2020 to August 2021. According to the guidelines of the American Thyroid Association Guidelines, overt hyperthyroidism was defined as a concomitantly suppressed serum thyroid-stimulating hormone (TSH) level and elevated serum free thyroxine (FT4) and/or free triiodothyronine (FT3) level [16]. Additionally, 100 age- and sex- matched healthy controls were recruited from physical examination center of Beijing Chao-Yang Hospital. The euthyroid healthy controls had no current or past thyroid dysfunction. Participants with the following conditions were excluded: age < 18 years, history of thyroid surgery, history of using thyroid drugs or systemic corticosteroids, history of using glucose- and lipid-lowering drugs, pregnancy, lactation, anemia, cancer, subacute thyroiditis, liver disease, chronic renal disease, severe cardiovascular or cerebrovascular diseases, current infectious conditions, psychiatric and neurological diseases. This study was approved by the Ethics Committee of Beijing Chao-yang Hospital. All enrolled subjects signed written informed consent.

### Anthropometric and biochemical measurements

All participants underwent anthropometric examinations, including age, sex, height, and body weight, by the same trained team. Body mass index (BMI) was calculated as body weight divided by height squared (kg/m^2^). After at least 12 h of overnight fasting, peripheral venous samples were collected in the morning for laboratory tests. FT3, FT4, and TSH were evaluated by electrochemiluminescence immunoassay using an Abbott Architect i2000 (Abbott Diagnostics, Abbott Park, IL, USA) as previously described [17]. Total cholesterol (TC), triglyceride (TG), high-density lipoprotein cholesterol (HDL-C), low-density lipoprotein cholesterol (LDL-C), and fasting blood glucose (FBG) were determined by an autoanalyzer (Hitachi 747, Roche Diagnostics, Germany). Fasting insulin (FINS) was detected by the chemiluminescence method (Dimension Vista, Siemens Healthcare Diagnostics). Serum Metrnl levels were measured using ELISA kits (R&D Systems, Minneapolis, MN, USA). The homeostasis model assessment–insulin resistance (HOMA-IR) was calculated as follows: FBG (mmol/L) × FINS (mIU/L)/22.5 [18]. The estimated glomerular filtration rate (eGFR) was calculated by the Chronic Kidney Disease Epidemiology Collaboration (CKD-EPI) equation [19].

### Statistical analysis

IBM SPSS 26.0 (IBM Corp., Armonk, New York, USA) and GraphPad Prism 9.0 (Inc, CA, USA) were used for the statistical analysis. The Kolmogorov–Smirnov test was conducted to assess the distribution of continuous variables. Normally distributed continuous variables were expressed as mean ± standard deviation (SD) and compared using unpaired Student’s t-test, and those continuous skewed distributed variables were expressed as median (upper and lower quartiles) and compared using Mann–Whitney U test. Categorical variables were expressed as number (%), and the Chi-square test was used to compare groups. The linear trend of hyperthyroidism proportion across the tertiles of Metrnl was accessed by the Cochran Armitage trend test. The correlations of serum Metrnl levels with FT3, FT4, TSH, BMI, TC, LDL-C, and TG were performed using Spearman correlation analysis. Linear regression models were used to explore the association of serum Metrnl concentrations with thyroid function parameters. In addition, logistic regression models were conducted to estimate the relationship between serum Metrnl and hyperthyroidism. The variables that were considered clinically relevant or showed a significant relationship in correlation analyses, as well as that without collinearity were selected for adjustment. Model 1 was unadjusted; Model 2 was adjusted for age, sex, and BMI; Model 3 was further adjusted for LDL-C, TG, HOMA-IR, and eGFR. A two-tailed *P* < 0.05 was considered statistically significant.

## Results

### Serum Metrnl levels in patients with hyperthyroidism

**Fig. 1:**
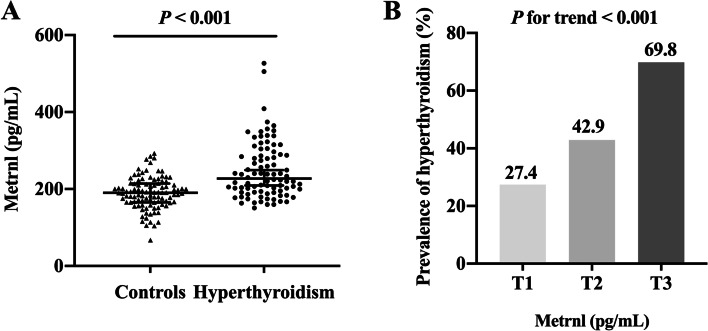
Metrnl and hyperthyroidism. **A** Comparison of serum Metrnl levels in subjects with and without hyperthyroidism. **B** The prevalence of hyperthyroidism across tertiles of serum Metrnl levels. Data were expressed as median (upper and lower quartiles) or proportion (%). *P* for trend from test for linearity

The baseline characteristics of subjects with overt hyperthyroidism and healthy controls are presented in Table 1. The mean age of the hyperthyroid patients was 40.28 ± 12.96 years. Compared with the controls, patients with hyperthyroidism had higher levels of FT3, FT4, and eGFR, and lower levels of TSH, BMI, TC, HDL-C, and LDL-C (all *P* < 0.001). Nevertheless, there were no significant differences in age, sex, TG, FBG, FINS, and HOMA-IR between the two groups. Of note, subjects with hyperthyroidism had higher levels of circulating Metrnl than the controls (*P* < 0.001, Fig. 1A).

**Table 1:** Clinical characteristics of participants with and without hyperthyroidism

Variable	Controls	Hyperthyroidism	***P***
	*n* = 100	*n* = 88	
Sex, male, n (%)	18 (18.0)	20 (22.7)	0.421
Age, years	40.89 ± 10.99	40.28 ± 12.96	0.729
BMI, kg/m^2^	24.23 ± 4.11	21.94 ± 3.43	** < 0.001**
TC, mmol/L	5.13 ± 0.94	3.52 ± 0.58	** < 0.001**
HDL-C, mmol/L	1.38 ± 0.37	1.14 ± 0.25	** < 0.001**
LDL-C, mmol/L	3.26 ± 0.99	1.94 ± 0.60	** < 0.001**
TG, mmol/L	1.14 (0.81, 1.93)	1.13 (0.83, 1.50)	0.239
FBG, mmol/L	5.04 (4.73, 5.34)	5.11 (4.72, 5.61)	0.308
FINS, uIU/mL	9.3 (6.5, 13.4)	10.9 (7.4, 15.9)	0.139
HOMA-IR	2.24 (1.39, 3.38)	2.59 (1.64, 3.50)	0.182
eGFR, mL/min/1.73 m^2^	111.3 ± 12.88	129.3 ± 17.06	** < 0.001**
FT3, pg/mL	3.28 (3.03, 3.47)	14.57 (8.92, 20.00)	** < 0.001**
FT4, ng/dL	1.26 (1.15, 1.38)	4.50 (2.90, 6.31)	** < 0.001**
TSH, μIU/mL	1.88 (1.35, 2.50)	0.01 (0.01, 0.01)	** < 0.001**
Metrnl, pg/mL	190.3 (165.4, 214.4)	227.2 (192.3, 289.2)	** < 0.001**

Data were expressed as the mean ± SD or median (interquartile range) unless stated otherwise

*BMI* body mass index, *TG* triglycerides, *TC* total cholesterol, *HDL-C* high-density lipoprotein cholesterol, *LDL-C* low-density lipoprotein cholesterol, *FBG* fasting blood glucose, *FINS* fasting insulin, *eGFR* estimated glomerular filtration rate, *FT3* free triiodothyronine, *FT4* serum free thyroxine, *TSH* thyroid-stimulating hormone, *Metrnl* Meteorin-like

Bold indicates *P* value < 0.05

### Correlation of serum Metrnl level with thyroid function parameters

**Fig. 2:**
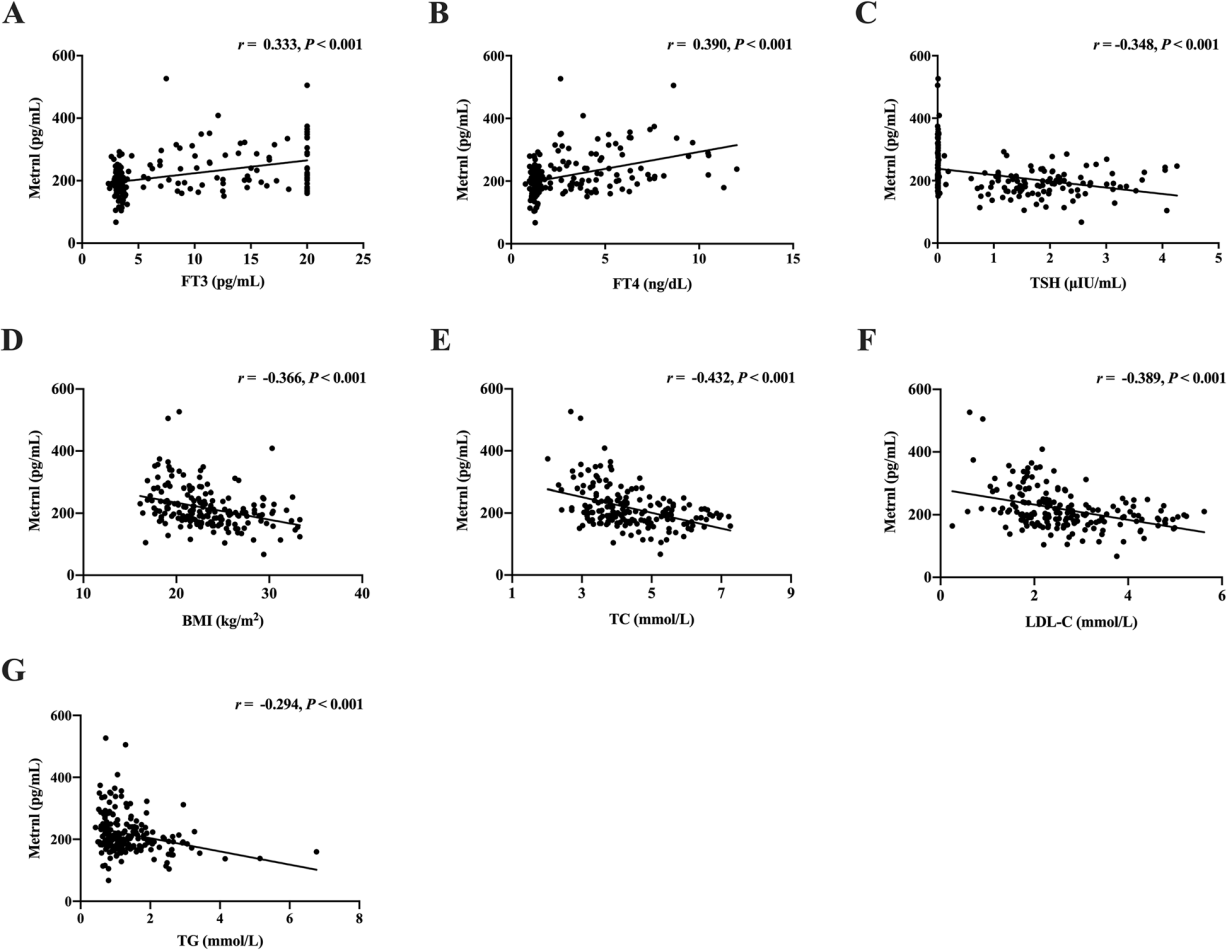
Correlation of serum Metrnl levels with **A** FT3, **B** FT4, **C** TSH, **D** BMI, **E** TC, **F** LDL-C, and **G** TG. Spearman’s correlation was performed for analysis due to the skewed distribution of Metrnl

As shown in Fig. 2, circulating Metrnl levels were positively correlated with FT3 (*r* = 0.333, *P* < 0.001) and FT4 (*r* = 0.390, *P* < 0.001), negatively correlated with TSH (*r* = -0.348, *P* < 0.001), BMI (*r* = -0.366, *P* < 0.001), TC (*r* = -0.432, *P* < 0.001), LDL-C (*r* = -0.389, *P* < 0.001), and TG (*r* = -0.294, *P* < 0.001) in all participants. Further linear regression analyses indicated that after full adjustment for confounding factors (model 3, Table 2), each 1 unit increase in serum Metrnl level was independently associated with FT3 (*β* = 0.324, *P* = 0.001), FT4 (*β* = 0.293, *P* = 0.001), and TSH (*β* = -0.234, *P* = 0.006).

**Table 2:** Linear regression analysis for the correction between serum Metrnl levels and thyroid function parameters

	Model 1		Model 2		Model 3	
Variables	Standardized *β (*95% CI*)*	*P*	Standardized *β (*95% CI*)*	*P*	Standardized *β (*95% CI*)*	*P*
FT3	0.407 (0.275, 0.539)	** < 0.001**	0.332 (0.199, 0.465)	** < 0.001**	0.324 (0.140, 0.508)	**0.001**
FT4	0.406 (0.274, 0.538)	** < 0.001**	0.324 (0.189, 0.459)	** < 0.001**	0.293 (0.117, 0.468)	**0.001**
TSH	-0.363 (-0.498, -0.229)	** < 0.001**	-0.303 (-0.436, -0.169)	** < 0.001**	-0.234 (-0.400, -0.070)	**0.006**

Metrnl was log transformed for analysis. Bold indicates *P* value < 0.05. Model 1: unadjusted; Model 2: adjusted for age, sex, and BMI; Model 3: adjusted for sex, age, BMI, LDL-C, TG, HOMA-IR, and eGFR

### Association of serum Metrnl levels with hyperthyroidism

The proportions of hyperthyroidism were progressively higher across Metrnl tertiles (*P* for trend < 0.001, Fig. 1B). As shown in Table 3, before adjusting for confounders, the OR (95% CI) of the highest Metrnl tertile was 6.130 (2.824–13.31) for hyperthyroidism compared with the lowest tertile. After further adjustment for age, sex, and BMI in model 2, a higher serum Metrnl concentrations remained significantly associated with hyperthyroidism. Furthermore, the relationship between elevated Metrnl levels and hyperthyroidism was still significant even after full adjustment (*P* for trend < 0.05, model 3). Meanwhile, per one unit increase in circulating Metrnl level was associated with hyperthyroidism in the fully adjusted model 3 (OR 1.021, 95%CI 1.007–1.036).

**Table 3:** Logistic regression analysis for the association between serum Metrnl levels and hyperthyroidism

	**OR (95% CI)**		
	**Model 1**	**Model 2**	**Model 3**
Per 1 unit increase	1.021 (1.013, 1.028)	1.019 (1.011, 1.027)	1.021 (1.007, 1.036)
Tertiles			
Tertile 1	Ref	Ref	Ref
Tertile 2	1.985 (0.939, 4.197)	1.709 (0.792, 3.688)	1.801 (0.530, 6.120)
Tertile 3	6.130 (2.824, 13.31)	4.878 (2.122, 11.22)	5.906 (1.430, 24.39)
*P* for trend	** < 0.001**	**0.001**	**0.047**

Bold indicates *P* value < 0.05. Model 1: unadjusted. Model 2: adjusted for age, sex, and BMI. Model 3: age, sex, BMI, LDL-C, TG, HOMA-IR, and eGFR

## Discussion

In this study, we identified that circulating Metrnl concentrations were significantly elevated in patients with hyperthyroidism. In addition, increased serum Metrnl levels were independently associated with hyperthyroidism. These findings provide insight into the clinical implication of Metrnl in hyperthyroid patients.

Metrnl is a newly discovered adipomyokine that beneficially affects body metabolism and thermogenesis. Rao et al. initially reported that overexpressing muscle-specific PGC-1α significantly increased the expression and secretion of Metrnl in mice [13]. Furthermore, Metrnl is produced in the skeletal muscle after exercise and in adipose tissue upon acute exposure to cold, respectively, and is present in the circulation [13]. Recent studies also revealed that exogenous Metrnl treatment modulated not only adipose tissue browning but also muscle growth and metabolism [13, 15, 20]. Since its discovery, many studies have been performed to examine the associations of circulating Metrnl with metabolic factors in various diseases. Several investigations have reported that circulating Metrnl is inversely correlated with body weight, BMI, and visceral fat area, as well as increased after bariatric surgery [21, 22]. Consistently, we also observed that serum Metrnl levels were significantly and negatively associated with BMI. In addition, exercise-induced muscle and plasma Metrnl effectively reduced fat accumulation in HFD-induced obese mice, suggesting that Metrnl appears to be a candidate for treating obesity [23]. However, it is not yet clear exactly how acute exercise triggers Metrnl secretion and, more importantly, what is the full physiological role of Metrnl actions in humans. Emerging research indicated that, at least in part, Metrnl may regulate adipose tissue browning and energy homeostasis.

TH, including thyroxine (T4) and its active form triiodothyronine (T3), plays important roles in modulating basal metabolism and thermogenesis. Based on the apparent similarity in the effects of TH and Metrnl on metabolism, we hypothesized that thyroid function could be directly or indirectly linked to Metrnl modulation, or vice versa, circulating Metrnl could affect the thyroid. To date, limited information is available on the relationship between thyroid dysfunction and Metrnl. In the present study, we identified that serum Metrnl levels were significantly elevated in patients with hyperthyroidism. However, the only study observed decreased serum Metrnl levels in patients with Graves' disease, which is inconsistent with our findings [24]. When comparing this result with ours, several aspects must be considered. First, the metabolic characteristics of subjects, such as glycemic parameters and lipid profile, may be different from ours. Previous studies have found that circulating Metrnl levels are associated with lipid profile [25], serum glucose and insulin resistance [26], which may affect serum Metrnl levels. Second, BMI was also not evaluated in their study, and the effect of BMI on Metrnl may also influence the result. Additionally, to explore the relationship between serum Metrnl concentrations and hyperthyroidism more directly, we further performed linear regression models adjusted for potential confounders and still found that serum Metrnl levels were positively correlated with FT3 and FT4 and negatively correlated with TSH.

It is well known that increased energy expenditure, weight loss and reduced cholesterol levels are characteristics of hypermetabolic state induced by excess TH in hyperthyroidism [5]. A major target of TH is adipose tissue. In brown adipocytes, TH promotes adaptive thermogenesis through increasing the expression of UCP-1 and PGC-1α [9]. Additionally, BAT contains highly expressed type 2 deiodinase (DIO2), which converts T4 to active T3 [27]. During cold exposure, BAT is activated via the DIO2 pathway, which leads to increased production of T3, expression of thermogenesis-related genes, and acceleration of mitochondrial respiration [28]. On the other hand, TH also stimulates WAT browning/beiging by increasing mitochondrial biogenesis and UCP-1 expression [29]. Hyperthyroid mice exhibited an increased expression of thermogenic genes in the WAT [30]. Moreover, administration of T4 or T3 could induce browning of WAT in rodent and humans [31]. Of note, Rao et al. reported that increasing circulating levels of Metrnl stimulated adipose expression of thermogenic genes, including UCP-1 and DIO2 [13]. Thus, it is not surprising that serum Metrnl levels were elevated in hyperthyroidism and positively correlated with FT3 and FT4 in our study.

Skeletal muscle is also an important target of TH action. TH exerts important effects on muscle contractile function, myogenesis, muscle regeneration, and energy metabolism [32]. Most patients with overt hyperthyroidism have clinically changes in skeletal muscle mass and function [33]. Additionally, T4-induced hyperthyroidism in mice exhibited increased muscle fatigue fatigability [34]. Besides adipose tissue, muscle is another major tissue producing systemic Metrnl. Recent study indicated that Metrnl was a vital regulator of muscle regeneration, and administration of recombinant Metrnl facilitated skeletal muscle repair via the Stat3/IGF-1 myogenic pathway [20]. Thus, another explanation for elevated serum Metrnl levels in patients with hyperthyroidism might be attributed to a protective compensatory response to muscle damage. Certainly, more research on the physiological regulation of Metrnl is warranted in the not-too-distant future.

In addition, TH directly and indirectly regulates cholesterol production, lipolysis and fatty acids β-oxidation. Excess TH stimulates the transcription of the LDL receptor gene, resulting in increased reverse transport of cholesterol to the liver for elimination. Furthermore, increased skeletal muscle metabolism and lipid oxidation were observed in patients with hyperthyroidism [35]. In our study, we found that serum Metrnl was negatively correlated with TC, LDL-C, and TG. Of note, previous studies have shown that deficiency of adipose tissue Metrnl exacerbated hypertriglyceridemia, whereas adipose tissue-specific overexpression of Metrnl attenuated hypertriglyceridemia in HFD-induced animal models [36]. Systemic administration of Metrnl alleviated lipid-induced inflammation and induced fatty acids oxidation by AMPK or PPARγ signaling in skeletal muscle [15]. Therefore, elevated circulating Metrnl levels in hyperthyroid patients may partially mediate TH actions on thermogenesis, lipid metabolism and energy homeostasis.

There are several limitations to this study. Firstly, the sample size was relatively small, and the cross-sectional design could not establish a causal relationship between Metrnl and hyperthyroidism. Secondly, changes in serum Metrnl levels after treatment in patients with overt hyperthyroidism were not measured. Thirdly, thyroid-related antibodies, body fat percentage, and muscle mass were not measured, which might hamper the power of our study. In addition, it was not to exclude other potential confounders, especially exercise and cold exposure. Lastly, our study included only Chinese people, so the generalizability of our results might be a concern. Given the above limitations, the present results still need to be further confirmed by longitudinal prospective studies in multi-ethnic populations.

## Conclusions

In conclusion, compared with healthy controls, serum Metrnl levels were significantly elevated in hyperthyroid patients and were independently associated with hyperthyroidism. Our findings provide clinical evidence for the significance of thyroid hormones in their interactions with Metrnl, while future investigations are warranted to confirm the underlying mechanism.

## Data Availability

The datasets used and/or analyzed during the current study available from the corresponding author on reasonable request.

## Abbreviations

Metrnl: Meteorin-like
TH: Thyroid hormone
TR: Thyroid hormone receptor
WAT: White adipose tissue
BAT: Brown adipose tissue
UCP1: Uncoupling protein 1
PGC‐1α: Peroxisome proliferator‐activated receptor gamma coactivator 1‐alpha
HFD: High fat diet
BMI: Body mass index
HOMA-IR: Homeostasis model assessment–insulin resistance
eGFR: Estimated glomerular filtration rate
DIO2: Type 2 deiodinase
